# Supplementary figures and images for: GEOlimma: differential expression analysis and feature selection using pre-existing microarray data
Source: BMC Bioinformatics. 2021 Feb 3;22:44. doi: 10.1186/s12859-020-03932-5 (PMC7860207; doi:10.1186/s12859-020-03932-5)

# TopDE plot

Top DE

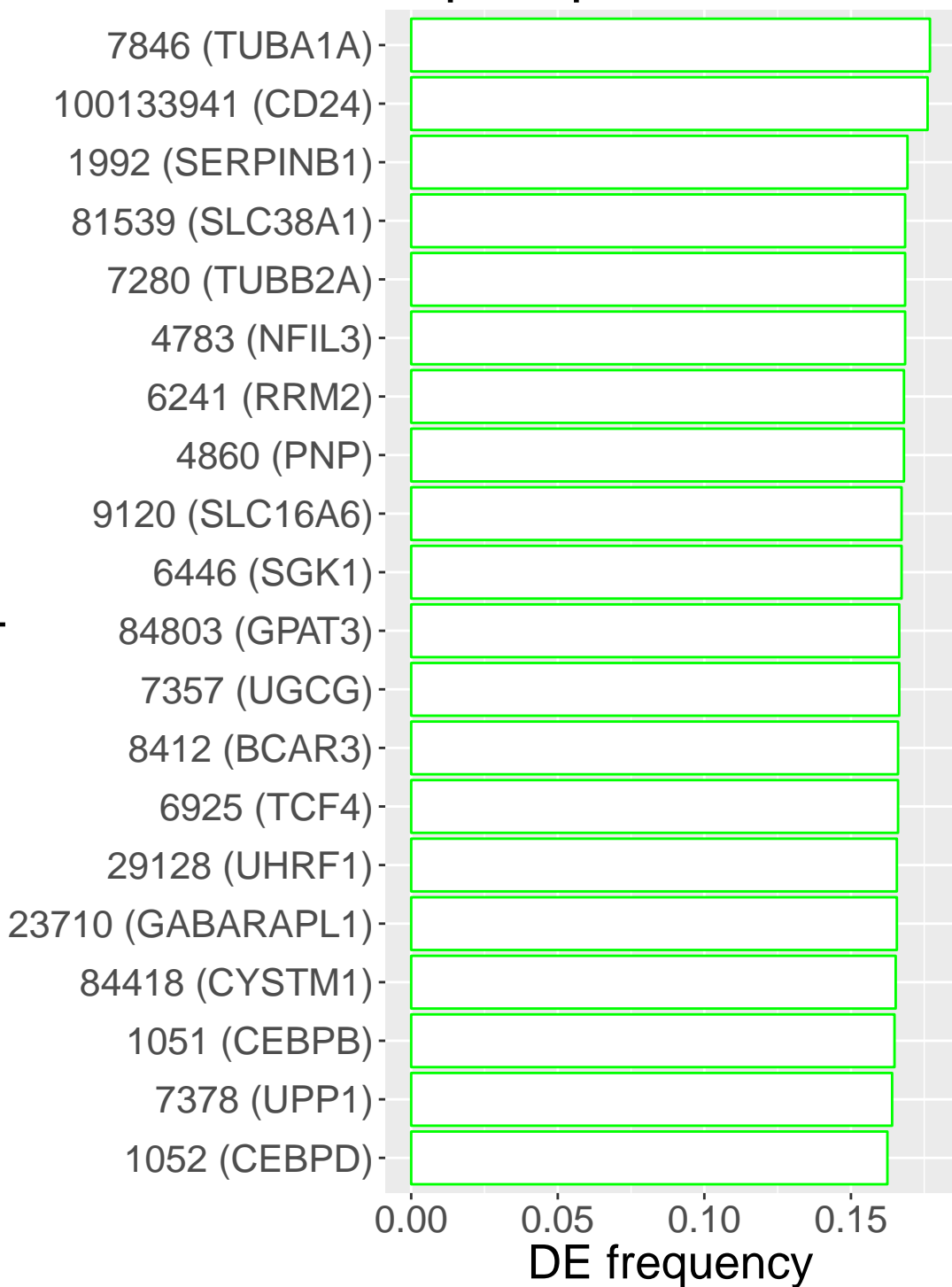

Supplement: Supplementary file 1 — Additional file 1. Barplot of top genes with high DE prior probabilities. [file 12859_2020_3932_MOESM1_ESM.pdf]

**A**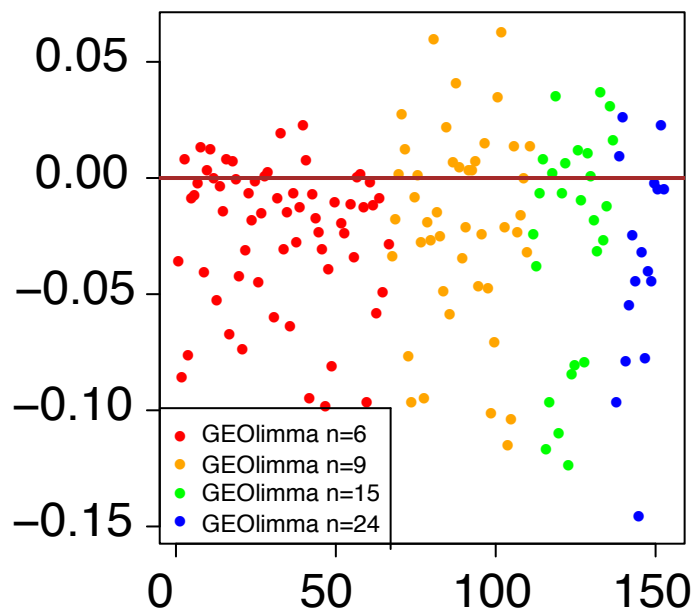**B**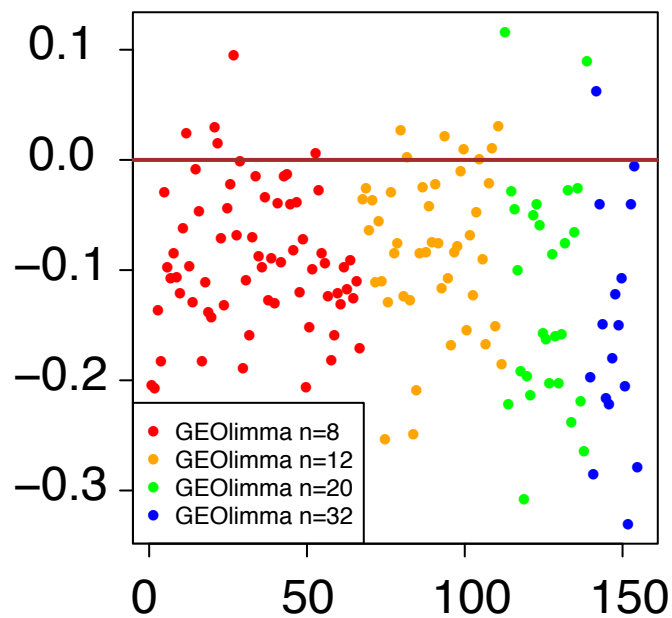**C**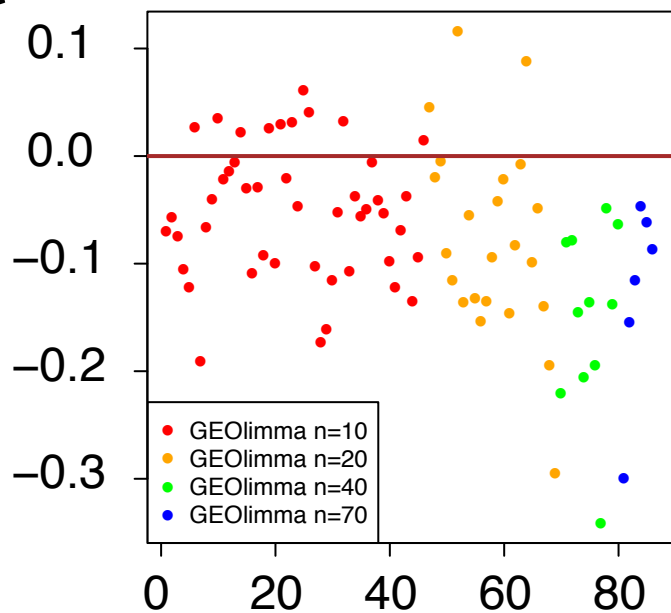**D**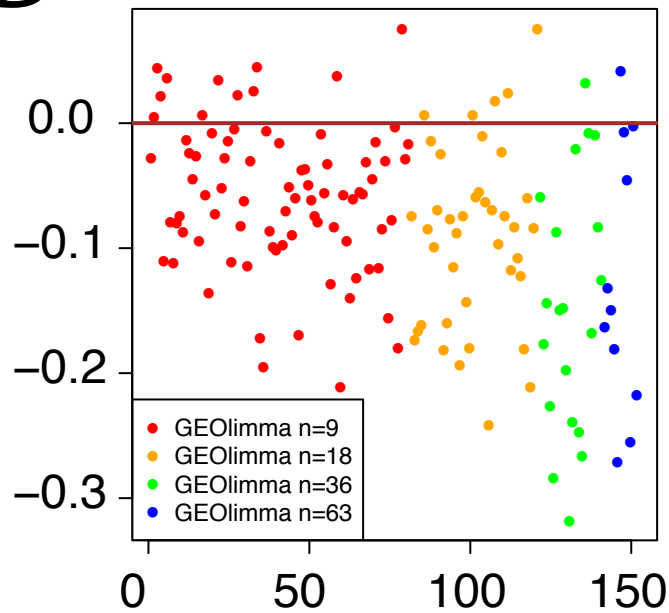

Supplement: Supplementary file 2 — Additional file 2. The B score order change between GEOlimma and Limma for the 20 most differentially expressed genes in the A) Asthma vs Non-asthma comparison, B) Nonleukemia vs AML comparison, C) Nonleukemia vs MDS comparison, D) AML vs MDS comparison. [file 12859_2020_3932_MOESM2_ESM.pdf]

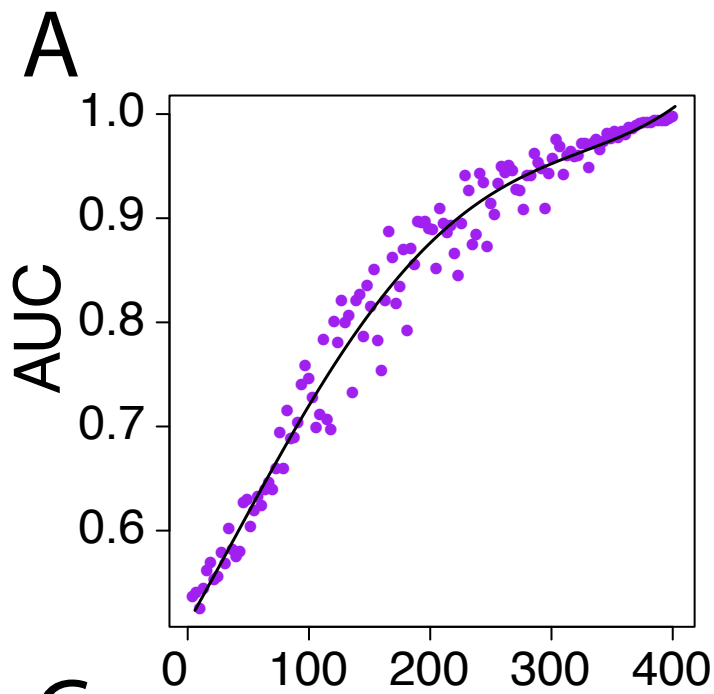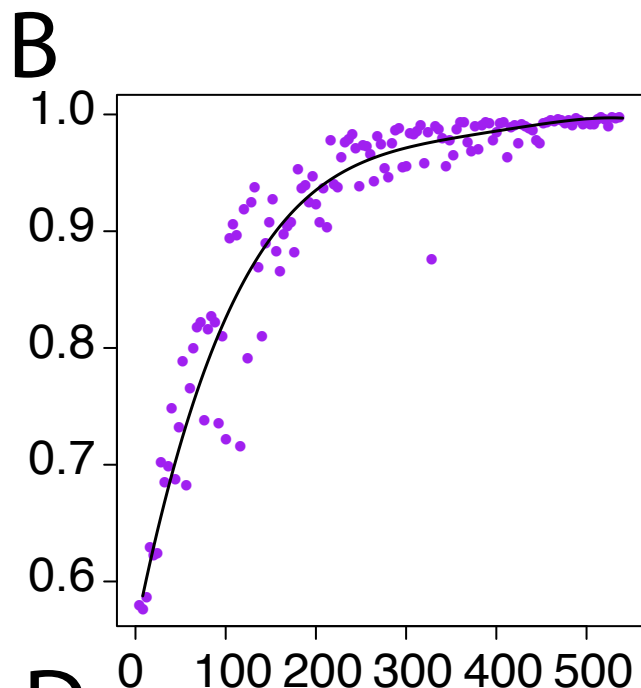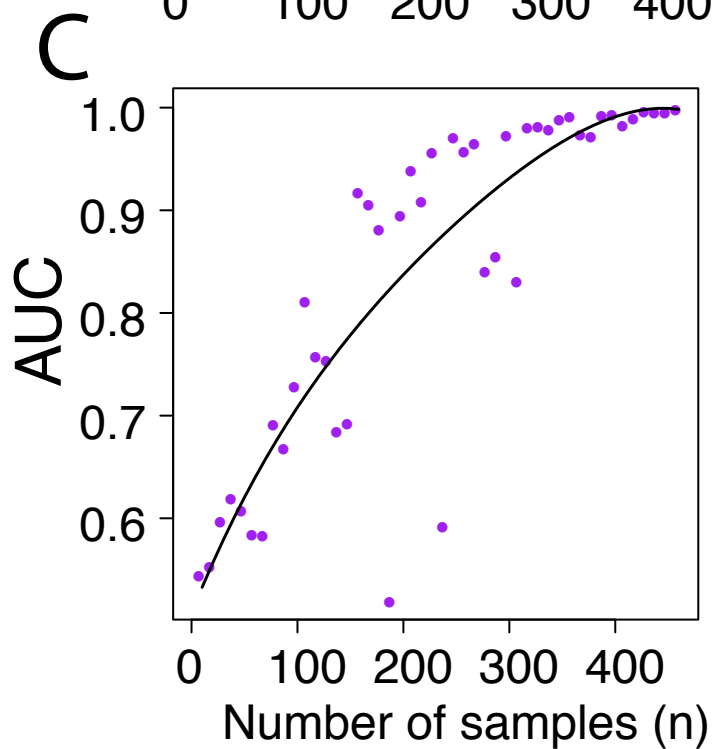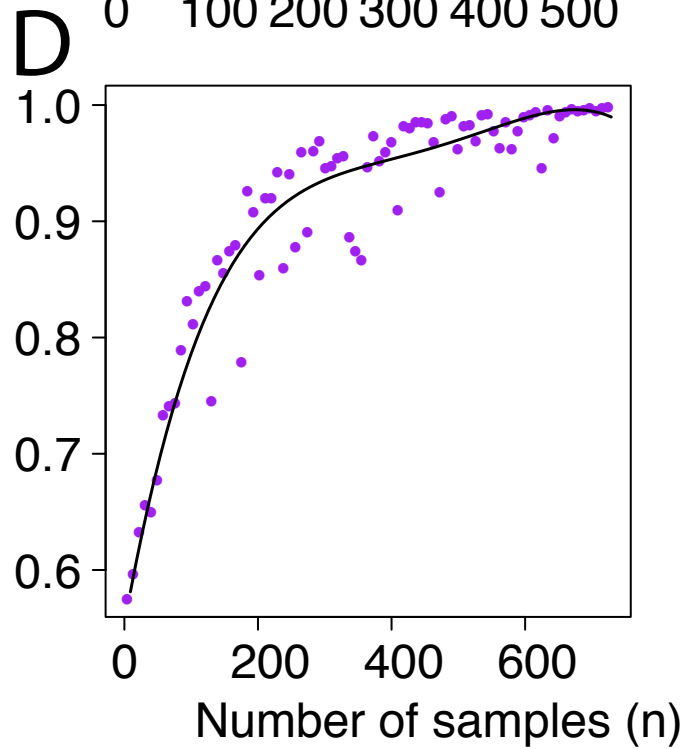

Supplement: Supplementary file 3 — Additional file 3. Percentage of genes in common from applying Limma and GEOlimma to 10 random data subsets across four differential expression comparisons. The x-axis indicates the number of top selected DE genes, while the y-axis indicates Percentage of genes in common. [file 12859_2020_3932_MOESM3_ESM.pdf]

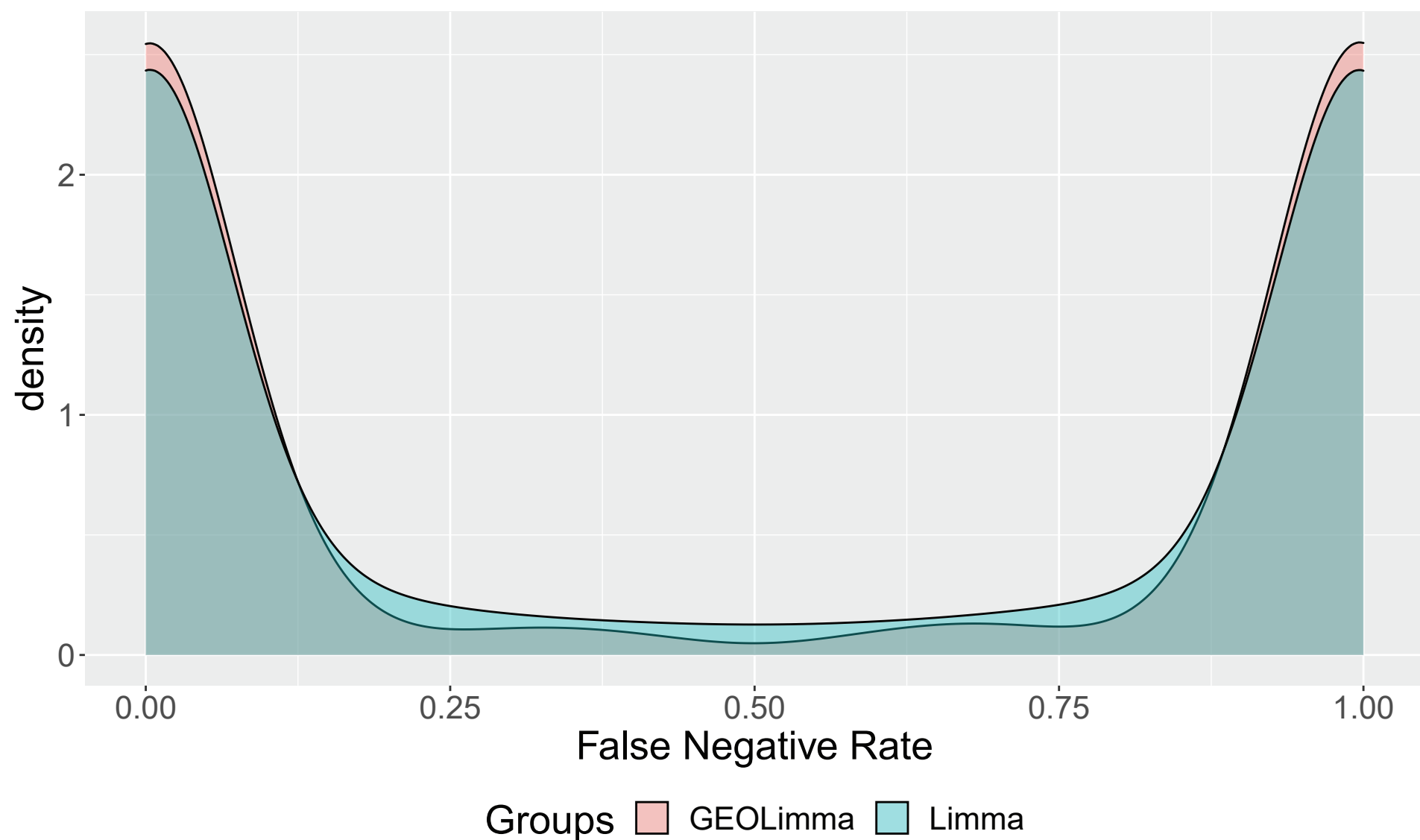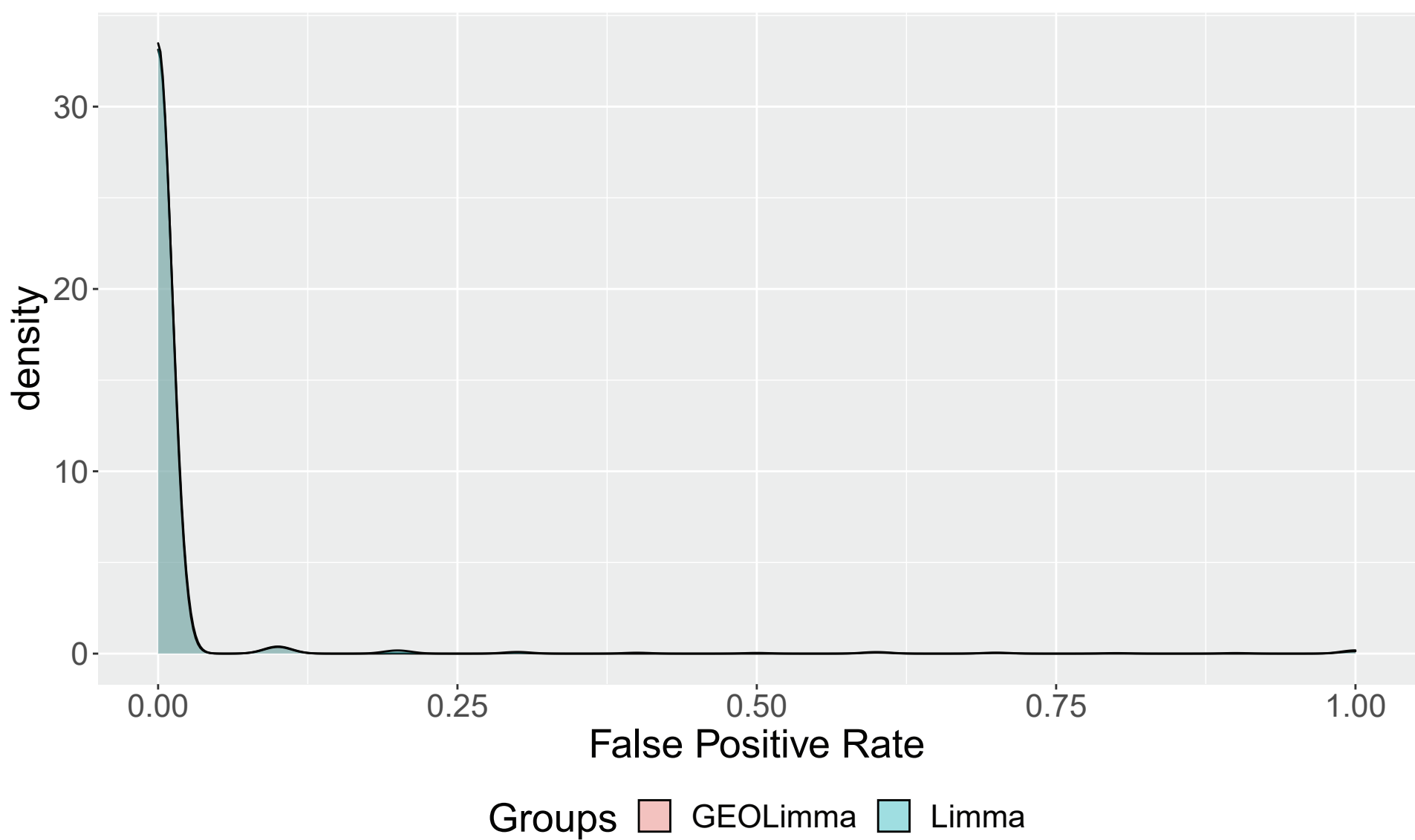

Supplement: Supplementary file 4 — Additional file 4. Gene-wise false positive rate (FPR) and false negative rate (FNR) distributions for Limma and GEOlimma resulting from a simulation study. [file 12859_2020_3932_MOESM4_ESM.pdf]

**A**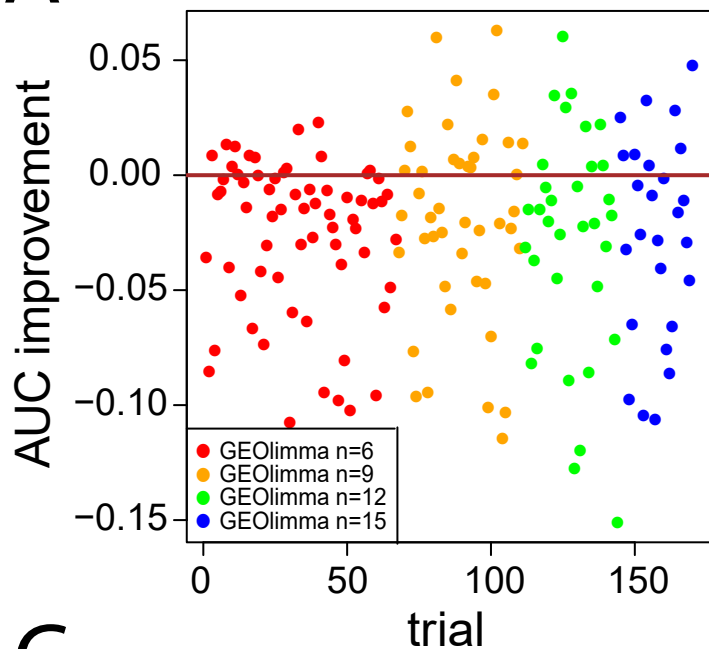**B**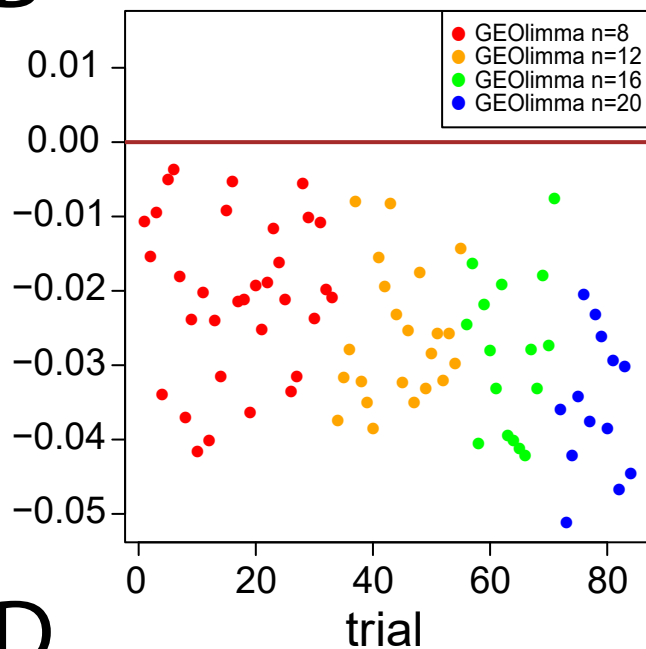**C**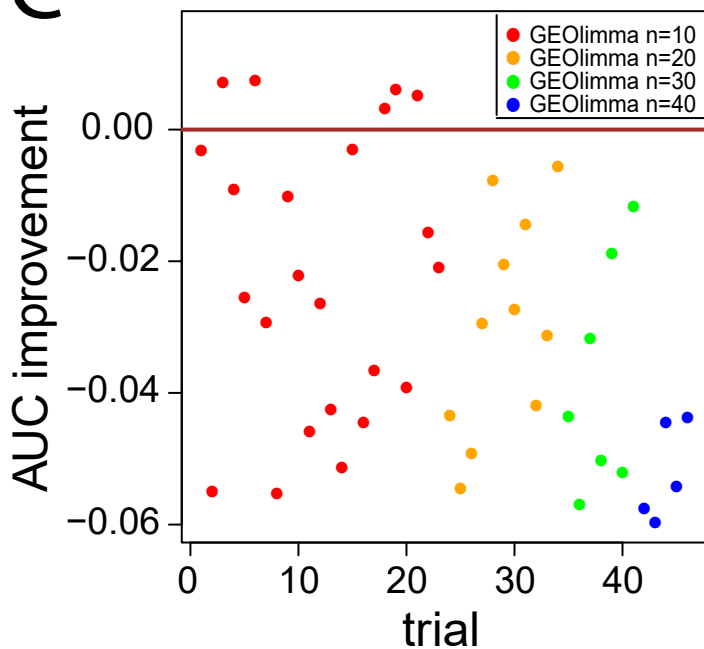**D**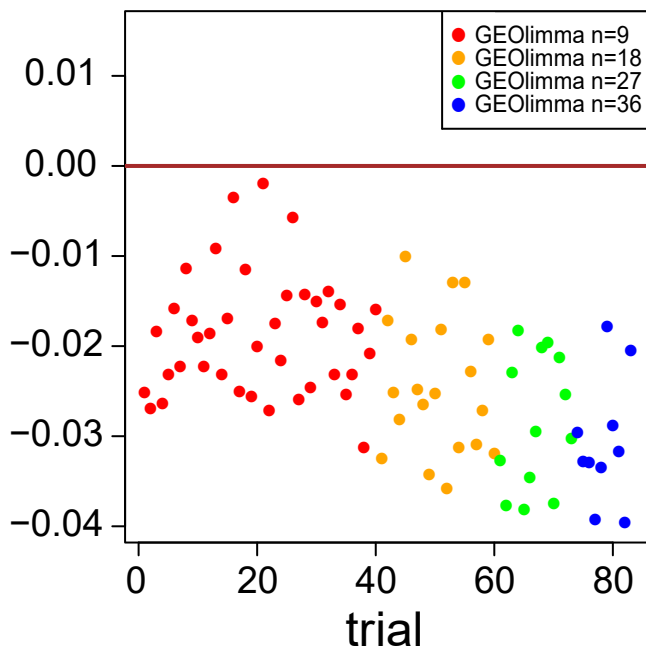

Supplement: Supplementary file 5 — Additional file 5. AUC improvement of GEOlimma with shuffled DE prior probabilities over Limma for the A) Asthma vs Non-asthma comparison, B) Nonleukemia vs AML comparison, C) Nonleukemia vs MDS comparison, D) AML vs MDS comparison. [file 12859_2020_3932_MOESM5_ESM.pdf]

**A**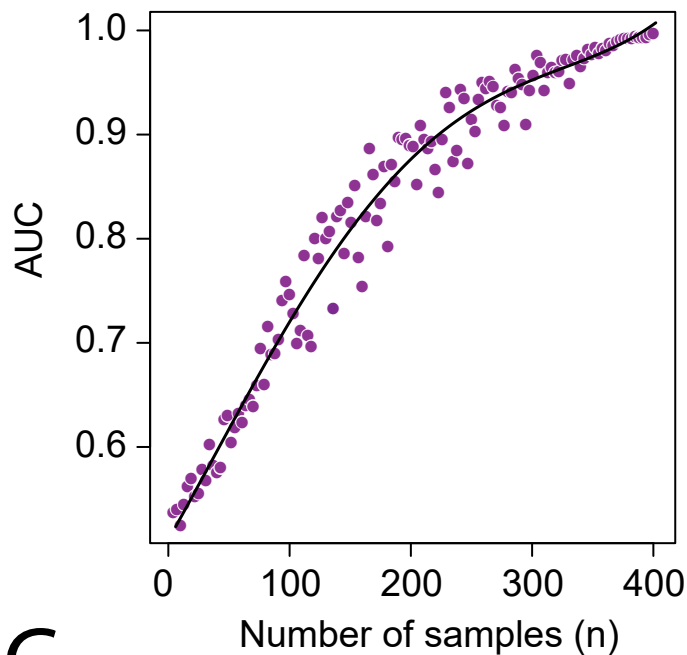**B**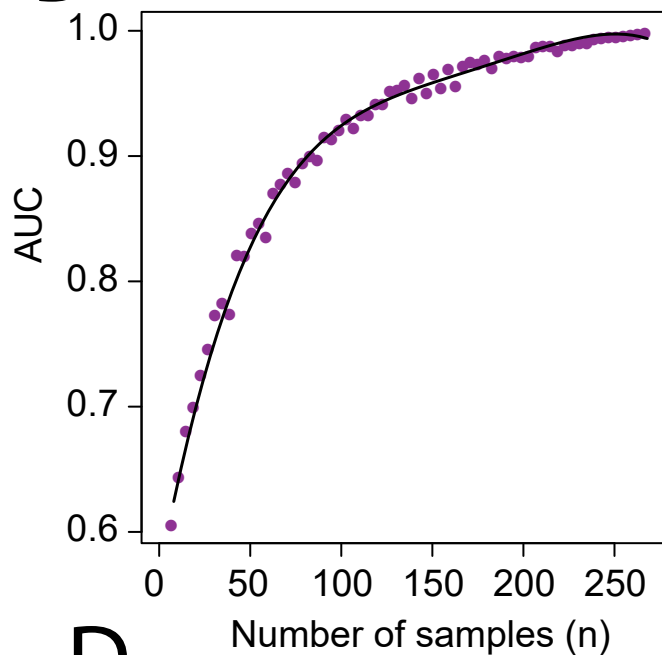**C**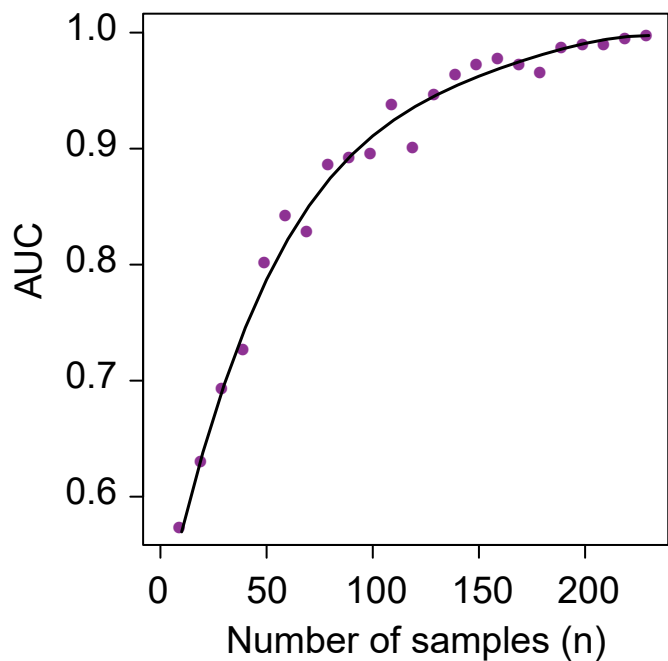**D**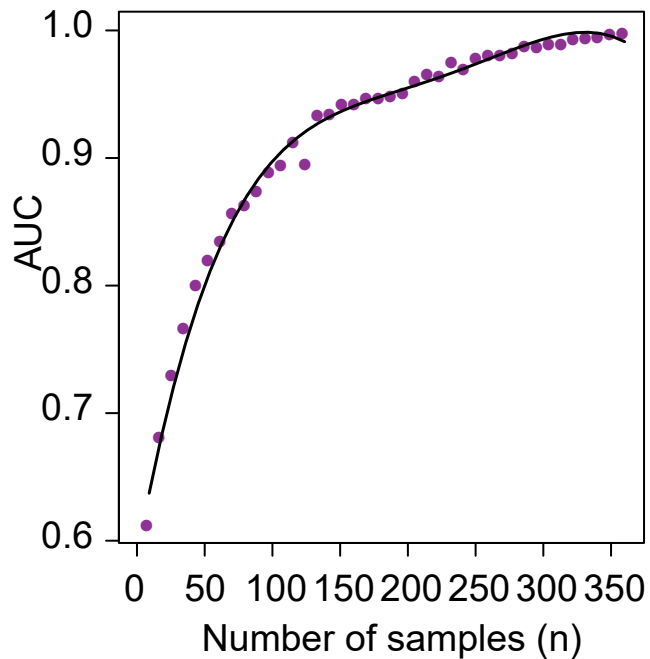

Supplement: Supplementary file 6 — Additional file 6. AUC standard curves for estimating effective sample sizes. A) Asthma vs Non-asthma comparison, B) Nonleukemia vs AML comparison, C) Nonleukemia vs MDS comparison, D) AML vs MDS comparison. [file 12859_2020_3932_MOESM6_ESM.pdf]
